# What Should Be Changed in Polish Neonatal Units in Order To Implement Family-centered Care?

**DOI:** 10.34763/devperiodmed.20192302.125130

**Published:** 2019-07-08

**Authors:** Magdalena Panek, Judene Mavrikis, Przemko Kwinta

**Affiliations:** 1 Zakład Zdrowia Matki i Dziecka, Wydział Nauk o Zdrowiu Uniwersytet Jagielloński Collegium Medicum w Krakowie, Krakowie Polska; 2 Klinika Chorób Dzieci Katedry Pediatrii, Uniwersytet Jagielloński Collegium Medicum w Krakowie, Krakowie Polska

**Keywords:** family-centered care, newborn, neonatal intensive care unit, role of parents, family-centered care, noworodek, intensywna terapia noworodka, rola rodzica

## Abstract

The progress that has been made in neonatology is associated with an increasing number of painful procedures constantly being performed on the neonate. Additionally, prolonged hospitalization of premature neonates in NICUs isolates the family from their child. Parents may state that they do not have any parental feelings and cannot communicate with their newborns. The FCC (Family-Centered Care) initiative responded to emerging reports about the adverse consequences ensuing from the lack of parental access to hospitalized children. The FCC should be understood as care based on partner relations between families and health professionals, which is supposed to lead to health and well-being for both the children and their parents. The FCC should become standard practice in all neonatal intensive care units.

## Streszczenie

Postęp jaki dokonał się w neonatologii związany jest z coraz większą liczbą bolesnych lub stresujących zabiegów wykonywanych przy pacjencie. Dodatkowo, wielotygodniowy pobyt noworodka urodzonego przed fizjologicznym terminem porodu w oddziale intensywnej terapii może wiązać się z długotrwałą rozłąką z jego rodzicami. Taka sytuacja może wywoływać u rodziców poczucie słabej więzi ze swoim dzieckiem. Inicjatywa FCC ((Family-Centered Care – opieka zorientowana na rodzinę) stanowi odpowiedz na pojawiające się doniesienia mówiące o konsekwencjach związanych z pobytem dziecka w szpitalu bez możliwości kontaktu z rodzicami. Zgodnie z definicją, poprzez FCC należy rozumieć opiekę opartą na partnerskich relacjach między opiekunami, a pracownikami służby zdrowia, która ma prowadzić do zapewnienia zdrowia oraz dobrego samopoczucia zarówno dziecku jak i jego rodzicom. FCC powinno stanowić standardową praktykę, dzięki której możliwe jest osiągnięcie opieki na nowym, wyższym poziomie.

## Introduction

Parental presence during a child’s hospitalization has been documented throughout history. At the beginning of the 20th century children admitted to the hospital were not allowed to stay with their parents. Visiting hours were very restrictive and in some hospitals parental presence was permitted only once a week for half an hour. These rules applied to both children requiring short-term as well as long-term hospitalizations [1]. As an increasing number of works indicating the unfavorable effects of separating children from their parents were published, this approach began to change. In Poland, the most important legal acts that determine the rights of hospitalized children and their parents are: Patient Rights Act, European Charter of Patients’ Rights and European Charter of Children’s Rights. Since 1991, with the adoption of the act on health care institutions, a child who stays in hospital has both the right to be visited and the right to additional care, staying with a mother or father around the clock. Hospitals were obliged to coordinate a system within pediatric wards to allow children 24-hour contact with their parents [2].

The FCC (Family-Centered Care) initiative responded to emerging reports about the consequences connected with the lack of parental access to hospitalized children. It defines the way to care for sick children as based on cooperation between medical staff and the patient’s family and aims to strengthen the bond between the family and the child during and after treatment by providing multidisciplinary care and psychosocial support [3]. The four basic concepts of FCC are: dignity and respect, information sharing, negotiation, partnership and collaboration between staff and parents. There are an increasing number of studies showing that the introduction of FCC principles in neonatal intensive care units can affect the neonates’ development in a positive way [4].

The purpose of this article is to summarize the available information on the history and basic assumptions of the FCC. Moreover, the authors presented both some of the benefits associated with the introduction of FCC rules for the care for neonates and the barriers that hinder using this method during everyday practice.

## Neonatal intensive care unit

With the development of neonatology the survival rate of premature and severely ill newborns has been rapidly increasing. In 2015 the percentages of preterm live births overall and by gestational age (22-31 weeks and 32-36 weeks) amounted to 7.3%. [5]. The progress that has been made in neonatology is associated with an increasing number of painful procedures constantly being performed on the neonate. According to existing research, procedures can range from 12 to 6 in one day to more extreme situations where up to 62 procedures may be performed daily [6]. In addition, the daily functions of the NICU (such as the monitoring and use of different kinds of equipment) exposes the hospitalized neonate in the ward to excessive noise and light. Considering that the stay in the NICU can take weeks, there is an increase in constant exposure to unsettling and disruptive stimuli, which can raise the risk of future neurobehavioral problems in school-aged children [7].

The development of neonatology has been largely focused on the introduction of modern, innovative equipment [8]. However, a consequence to the stimuli made by these modern machines is a possible delay in the neonate child’s neurological development. The unique nature of neonate care requires that the recipient of care should not just be the neonate but the parents as well, which is often an ignored concept. This holistic approach to caring for a newborn and his/her family can be a double challenge for staff. It involves both manual dexterity, know-how regarding the use of specialized equipment, as well as cooperation with the parent who is in a stressful and completely unexpected situation. Already during the first moment after the child is admitted to the ward, parents struggle with such feelings as dread, confusion and fear. Negative emotions are associated not only with the child’s severe condition but also with the presence of unknown, noisy, and highly specialized equipment that surrounds their child. Riper showed that the parents whose child was in the intensive care unit were accompanied by both feelings of relief and reassurance as well as fear, anger, guilt, or loss of hope [9, 10]. In other studies, however, the presence of one’s newborn in the NICU was felt as inability to care for the child, as well as the inability for family members to understand and share the parent’s emotions. The parents who took part in the study emphasized the great need to be close to their newborn and have the possibility of getting to know the infant [11]. Furthermore, Carter et al., showed a higher risk of marriage breakdown and the emergence of financial difficulties in those families whose child was admitted to a neonatal intensive care unit [12]. Additional authors focused on the difficulties parents faced after their newborn had been discharged home. Long-term observation showed that at least in the initial period after leaving the hospital, parents experienced problems with taking care of their newborn. The situation was particularly aggravated in scenarios where for various reasons the possibility of contact with the neonate was limited. Parents became helpless in the face of simple problems that may be encountered at home, such as newborn crying or experiencing colic [13]. In 1960, Bowlby and Robertson drew attention to the negative consequences associated with the separation of mother and child due to hospital admission. In addition, they emphasized the unique role of a parent in caring for a sick child [14]. Numerous theories have developed in response to the emerging reports on the role of contact between parents and their hospitalized children, which changed the approach of medical staff to the presence of parents. FCC is one of the models of conduct for dealing with a neonate staying in the neonatal intensive care unit.

## History and main assumptions of family-centered care

The first specialized hospital focused on childcare opened in 1802 in Paris. Soon afterwards, subsequent hospitals began to appear: in 1834 in St. Petersburg, in 1837 in Vienna and in 1852 in London. Before the outbreak of the First World War, hospitals put a great emphasis on the emotional needs of children. Before 1920, nursing care focused not only on psychological problems but also on the social situation of children. After 1920, the approach to caring for hospitalized children changed. As these countries experienced the negative effects of war, new rules were followed regarding childcare in hospitals. At that time, both nursing and medicine were directed to focus on fighting against infectious diseases that were encountered on the battlefield. Hospital staff were not only looking for ways to manage existing diseases but also to find methods that would limit the spread of infection. One of these was to separate children from their parents. Increasingly often hospitals introduced bans on parental visits. In the period from the outbreak of the First World War until 1970, there were only a few hospitals that allowed the presence of parents. The number of such places was, however, insufficient to play an important role in the development of FCC and to change the approach to childcare [1, 15].

Over time, reports showing a minimal relationship between the incidence of infection in hospitals and the presence of parents were published. Meanwhile, psychiatrists conducted studies among adults who had experienced childhood hospitalizations that involved separation from their parents. In 1945 Renee Spitz described hospitalization as an “orphan disease” to signify the impact of hospitalization without the presence of a parent. Nevertheless, the Second World War had again changed the role of parental presence in caring for a hospitalized child. After this war, 1.5 million Polish children were orphaned, half-orphaned or abandoned. This situation was also characteristic for many other countries. Psychologists began to focus on the phenomenon related to the separation of families caused by the necessity of evacuation, resettlement, or loss of life. They were able to demonstrate the connection between this issue and the separation of children from their parents during hospitalizations [1, 2, 15].

Various models of childcare, such as parental participation in care, parental care, or partnership in care, continued to change the relationship of parents with their hospitalized children. Each of these was based on the presence of a parent at the child’s side during the entire stay in the hospital, parental childcare as well as taking part in the decision-making process related to the treatment of the child [17]. The concept of FCC was implemented for the first time in 1992 by the Institute for Patient and Family-Centered Care in the US (IPFCC) [18]. Since then, many new definitions have started to appear. One of these was created in 1995 by a team consisting of parents and health professionals under the leadership of the Maternal and Child Health Bureau (MCHB). Afterwards, in 2005, the concept and foundation of the FCC was improved on the basis of twenty years of dialogue between healthcare professionals and parents. According to the definition, the FCC should be understood as care based on partner relations between families and health professionals, which is supposed to lead to health and well-being for both children and their parents. This approach respects cultural differences, different traditions and skills represented by all of the participating members. The FCC should become standard practice in caring for all neonates in neonatal intensive care units.

The FCC’s basic assumptions are:

- Cooperation between medical staff and parents
- Patient care focused on the well-being of the child and his or her parents
- Common respect for skills and competences
- Building a sense of trust
- Open and objective communication
- The language and manner of providing information adapted to the level of knowledge and experience of the recipients
- Joint decision making about the care, treatment and further therapeutic process of the child
- Continued willingness to negotiate

Compliance with the basic FCC assumptions may result in:

- Considering the parents’ relationship with the child as something permanent
- Strengthening the bonds between parents and the child staying in the intensive care unit
- Respecting cultural differences and socioeconomic diversity
- Providing an individual, developmentally supportive model of neonatal care
- Developing a hospital policy founded on a friendly approach to families [19]

Another care model that is also important and worth implementing into neonatal care units is NIDCAP (Newborn Individualized Developmental Care and Assessment Program). NIDCAP is a form of developmental care in a NICU, which facilitates holistic support of the physical, mental and emotional development of newborns in the group of high-risk developmental disorders. It was created for the education and training of staff and parents in reading a neonate’s behavioral cues. According to this model, they learn how to adapt their care according to the individual needs of the newborn. The original assumption of the NIDCAP method is to take care of the parents and encourage them regarding the constant and active care of the child. This method also assumes modification of the NICU environment in order to limit non-physiological conditions that preponderate in the unit and which would adversely affect a child’s development. Additionally, an infant’s hospital space must be recognized as the infant’s and family’s immediate home in order to help adults grow in their role as competent parents. Furthermore, both methods (FCC and NICAP) emphasize the role of psychological care in neonatal units. In Poland, the need for such care was mentioned in recommendations published by the Polish Neonatal Society in 2015. Psychological care should have a long-term nature and not only include the period of hospitalization, but also the first years of a child’s life. In Poland, not all III level NICUs employ psychologists on a permanent basis, but more and more hospitals are introducing elements of individualized development care [20, 21, 22].

## FCC in neonatal intensive care –benefits, barriers

The importance of implementing FCC rules in neonatal units is emphasized because the hospitalization often lasts many weeks. Lack of or limited contact with a neonate for such a long time may result in the parents’ feeling they have lost control and promote incorrect parental attitudes. The parent becomes dependent on the medical staff and the equipment surrounding his or her neonate. In the classic model of neonatal care, the staff take on the duties of parents, spending more time with the neonate and consequently depriving families of the opportunity to interact. It should always be considered that the first years of life are extremely important for creating bonds between children and their parents; additionally, the neonatal period itself is associated with the intensive growth and development of the neonate, all which can be affected by a lack of parental presence. A newborn’s stay in an environment that is not aimed at supporting his or her development may result in negative health consequences both in childhood and in later life [19].

An increasing number of papers continue to put emphasis on the benefits of implementing FCC rules in the daily practices of the ward. The results of randomized trials carried out in a group of 110 newborns indicated that the FCC program seems to be effective in increasing maternal satisfaction and decreasing neonatal readmission [23]. O. Erdeve et al. compared a group of newborns who were allowed quality time in separate rooms with their mother with a group of those admitted to classical intensive care units, where there was little parental participation. They showed that the availability of single rooms is correlated with a lower number of rehospitalizations [24]. Ortenstrand showed that parental involvement in the care of infants in NICUs may reduce the total length of stay for infants born prematurely by an average of 5 days. [25]. Pineda et al. explored the relationship between NICU room type (private room and open room). They focused on the outcome of neurodevelopmental performance at the age of two years measured using the Bayley Scales of Infant and Toddler Development and concluded that infants from private rooms had lower language scores and lower motor scores. Their findings suggest that excessive noise and sound reduction can, in some cases, be detrimental to childhood development. More research is needed to determine the optimal NICU environment [26].

The most important aspects related to FCC in neonatal intensive care units include:

- The presence of a parent. The FCC approach assumes the constant presence of a parent with the child, which means that parents not only take care of the child but participate in appointments, painful procedures and other everyday events related to the functioning of the ward. A parent should be treated like a partner and not like a person visiting his or her neonate. This step also emphasizes the role of the parent in the use of non-pharmacological methods of pain relief. His or her presence during painful procedures may affect the sense of control and strengthen parental competence.
- Engaging the parent in care. It should be remembered that at the beginning parents may be too scared by the situation and be afraid of nursing their child. An important role here is played by a nurse who observes the reactions of the parents and gradually involves them in caring for the child. For this purpose, a parent’s participation card can be used (tab. I). The aim is to improve cooperation between medical staff and parents. The chart contains information about the level of parental involvement in performing individual procedures. The extent to which parents participate in individual procedures depends on the staff dealing with the newborn, the general condition of the child, and the availability of the parents. The chart should provide clear information for each subsequent person dealing with the newborn regarding the level of parental involvement and the need to explain specific activities.
- Communication with parents. On-going discussion about the condition of their newborn is necessary for parents to become partners in the treatment process and make decisions related to the methods of treatment or care. Parents should receive support by feeling that their observations regarding the newborns condition are an important element in the treatment process. They should be encouraged to attentively observe their newborn by asking questions related to specific behaviors (breathing method, feeding tolerance, the child’s sleep) [27].

**Table I:** Parent's participation chart in the care of a newborn (example procedures). Tabela I. Karta udziału rodzica w opiece nad noworodkiem (przykładowe procedury).

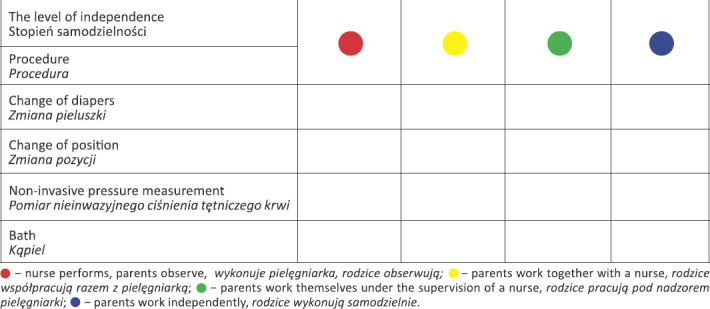

In addition to publications indicating the benefits of introducing FCC to neonatal departments, there are also reports pointing to both barriers and difficulties occurring when using this model of care. The barriers related to the introduction of FCC can be grouped as follows: difficulties in understanding FCC protocol and FCC research or financial problems. The former result from the fact that the majority of recommendations are based on how care should comply with FCC principles, without indicating specific solutions. An additional problem results from the lack of understanding the benefits that result from parental cooperation. Neither medical staff nor parents always understand what to expect from working together. An approach based on placing too high of a demand on a parent who is already facing a difficult situation may cause a negative perception of childcare. The second group concerns financial barriers: these come down to the belief that it is necessary to introduce changes that are primarily related with rebuilding the unit in order to create better housing conditions [4].

Additional reviews showed that health care professionals worried about losing their status. For some members of the therapeutic team, allowing parents to perform selected nursing activities may be associated with a sense of loss of control and position. In such situations it should be remembered that the implementation of FCC principles is not based on releasing duties. The FCC focuses on taking responsibility for both children and their parents. The role of medical staff in this case is double, it involves educating parents and being with them during procedures, as well as maintaining a continuous dialogue focused on common trust and understanding of needs [28].

## Summary

The basic right of every child is to be close to their parent. This right should also be respected with regard to newborns in neonatal intensive care units. FCC is an approach that can be successfully implemented in hospital wards, as it does not require major financial expenditures but is only associated with a change in the functioning and mentality of the ward. It is worth noting that management according to FCC rules leads to individualized care that is based on continuous observation of the child. Thanks to this, it is possible to provide care adapted to each newborn’s individual activity. The role of the staff is the gradual involvement of the parent in the care of their child, which is associated with a change in the attitudes towards parental roles in caring for children in intensive care units. FCC is a method based on a long history which can positively affect the current and further development of the child. It is a philosophy, not a one-time approach to parental presence in intensive care units. There is no data regarding the situation related to FFC practice in Polish neonatal units. Taking into account the benefits of using this method, it can be concluded that this model should be introduced in all neonatal units in Poland.
